# Community attitudes towards Amur tigers (*Panthera tigris altaica*) and their prey species in Yanbian, Jilin province, a region of northeast China where tigers are returning

**DOI:** 10.1371/journal.pone.0276554

**Published:** 2022-10-27

**Authors:** Ying Li, Joshua Powell, Aifen Jin, Hee Kyung Ryoo, Hailong Li, Puneet Pandey, Weihong Zhu, Dongwei Li, Hang Lee

**Affiliations:** 1 Tiger & Leopard Conservation Fund in Korea (KTLCF), Seoul, Republic of Korea; 2 Research Institute for Veterinary Science, College of Veterinary Medicine, Seoul National University, Seoul, Republic of Korea; 3 College of Geography and Ocean Science, Yanbian University, Yanji, China; 4 Institute of Zoology, Zoological Society of London, London, United Kingdom; 5 Department of Geography, UCL, London, United Kingdom; 6 Sustainable Conservation Network, Yanji, Jilin, China; 7 National Forestry and Grassland Administration Key Laboratory for Conservation Ecology in the Northeast Tiger and Leopard National Park, Hunchun, Jilin, China; 8 Hunchun Forestry Department, Hunchun, Jilin, China; Cheetah Conservation Fund, Namibia University of Science and Technology, NAMIBIA

## Abstract

Community attitudes towards large carnivores are of central importance to their conservation in human-dominated landscapes. In this study, we evaluate community attitudes and perceptions towards the Amur tiger (*Panthera tigris altaica*), Amur leopard (*Panthera pardus orientalis*) and bears (*Ursus thibetanus* and *Ursus arctos*), as well as their prey species, namely sika deer (*Cervus nippon*), roe deer and wild boar (*Sus scrofa*), in Yanbian Korean Autonomous Prefecture, Jilin province, northeast China. We surveyed 139 households and found that community members’ perceptions of large carnivores and their prey species were influenced by their predominant economic activities; their prior interactions with wildlife; their household income level; and whether they were either long-term residents of Yanbian or had migrated to the region from elsewhere in China. We recorded fairly neutral attitudes towards large carnivores among the communities we surveyed, but strongly negative attitudes were shown towards wild boar, particularly where respondents had lost agricultural products to crop raiding by wild boar. We recommend conservation stakeholders in northeast China utilise this finding to encourage support for large carnivore recovery and conservation by targeting messaging around the importance of the tiger as a key predator of wild boar in the ecosystem. Furthermore, our findings suggest that government provided compensation paid for cattle lost to large carnivore predation (notably, by tigers) may be helping to reduce animosity from cattle owners towards large carnivores. However, we also highlight that compensation for loss of livestock is therefore performing a useful role in mitigating human-wildlife conflict, that there are potentially unintended consequences of the current compensation program, for example it fails to dissuade livestock grazing in protected areas.

## Introduction

Large carnivores play an important ecological role [1], but have experienced widespread population declines as a result of human activities [2, 3]. Given the potential for conflict between humans and large carnivores [2, 4], the perception of local communities towards large carnivores can be a particularly important factor in their persistence or extirpation [5, 6].

The tiger (*Panthera tigris*) is one of the world’s largest extant carnivores [7]. Formerly widely distributed in Asia [8], tigers now inhabit just 6% of their historical range [9], with the total global population of wild tigers having declined to approximately 3500 animals by 2014 [8]. In 2010, the Global Tiger Recovery Program was launched by all 13 extant tiger range countries, with a commitment to double the number of wild tigers by 2022. Progress towards this commitment has seen limited success. Tiger numbers in some range countries have increased over the past 12 years, notably in Nepal [10], India [11] and northeast China [12],but have declined in other range countries in the same time period, for example, Malaysia [13].

Anthropogenic threats to tiger are of preeminent concern, both where this results in the intentional killing of tigers, such as the killing of tigers for the purpose of trade in their body parts or as a result of human-tiger conflict, as well as where this results in accidental fatalities, such as road kills traffic collisions between vehicles and tigers [13, 14]. As a result, engagement with community stakeholders is essential for ensuring the success of tiger conservation [15]. Conflict between humans and tigers may generate negative perceptions among local communities, particularly where it results in the loss of human life or livestock, which can lead to the retaliatory killings of tigers [16, 17]. Studies which seek to understand the influence of human-tiger interactions on community attitudes towards large carnivores and their prey species can help us to better understand these interactions and ensure the effective implementation of conservation strategies aimed at reducing conflict between humans and tigers[18–20].

While the tiger has been extirpated across much of China, the country’s northeast provinces of Jilin and Heilongjiang have a small and growing, Amur tiger (*Panthera tigris altaica)* population [21]. In 1998, the tiger population in Jilin province was believed to be as few as 7–9 individuals with a further 5–7 individuals in Heilongjiang [22], but the population size has steadily increased over the past two decades; between 2012–2014, 26 individual tigers were identified [12], while between 2013–2018, 55 tigers were identified in the broader northeast China region [21]. To aid this recovery, a series of protected areas have been established in order to protect suitable habitat, which might support future tiger populations. For example, in 2001, Jilin Hunchun Northeast Tiger Nature Reserve was established in Hunchun, Jilin province and in 2017, the Chinese government established the Northeast China Tiger and Leopard National Park (NTLNP) was established, covering an area of 14,600 km^2^ and with the aim of supporting the recovery of both Amur tiger and Amur leopard (*Panthera pardus orientalis*) populations. However, previous studies have identified human activities in and around these protected areas, such as livestock grazing in protected forest areas, as a major challenge for the objective of tiger recovery [23]. A substantial portion of the income of local communities comes from livestock grazing in tiger habitat, leading to potential human-tiger conflict [24]. Determining the factors which influence attitudes towards tigers and their prey species, as well as better understanding the needs of the local community, can help identify direct and indirect threats to tigers and provide suggested interventions which will have the support of those communities [25].

**Fig 1:**
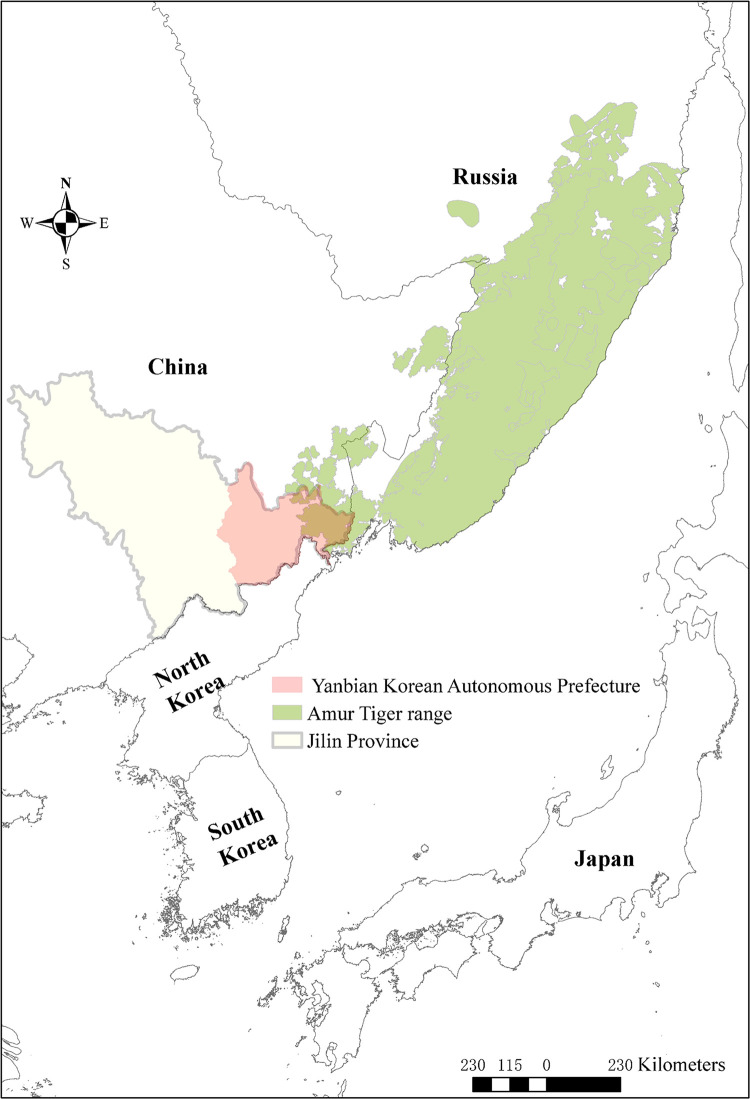
Map of the extant range of the Amur tiger in relation to Yanbian Korean Autonomous Prefecture, China. Amur tiger range was drawn based on IUCN, 2015 [8], and base layers were created through ArcMap 10.3 (ESRI, Redlands, USA).

Developing a better understanding of the perspectives and concerns of local communities may be particularly important in our study area, given that 70% of the designated Northeast China Tiger and Leopard National Park is located in a semi-autonomous region of China, Yanbian Korean Autonomous Prefecture, a semi-autonomous region of China, which shares land borders with the Democratic People’s Republic of Korea (North Korea) and Russia, constituting the majority of the Amur tiger’s current distribution in China [26] (Fig 1). 37% of Yanbian’s two million residents are part of the Korean Chinese ethnic minority, with their own traditions, customs, and identity, with the remainder being Han Chinese or belonging to other ethnic minority groups [27]. In 2019, the region had a GDP per capita of $5,000 USD, less than half of the national average for China [27], meaning that residents may potentially be more vulnerable to economic losses resulting from human-wildlife conflict.

In this study, we sought to investigate the attitudes of the local communities in this region towards tigers, other large carnivores including Amur leopards and bears, and the main prey animals of tigers, including roe deer (*Capreolus pygargus*), sika deer (*Cervus nippon*) and wild boar (*Sus scrofa*). We hypothesized that the local community would have different perceptions of different species and that a range of factors, including demographic characteristics, income level educational background, as well as prior depredation experience, would drive different attitudes towards these target species within the community. We first used factor analysis and cluster analysis to understand attitude structures, and applied regression modelling to check how the relevant factors influenced respondent attitudes.

## Material and methods

### Study area

**Fig 2:**
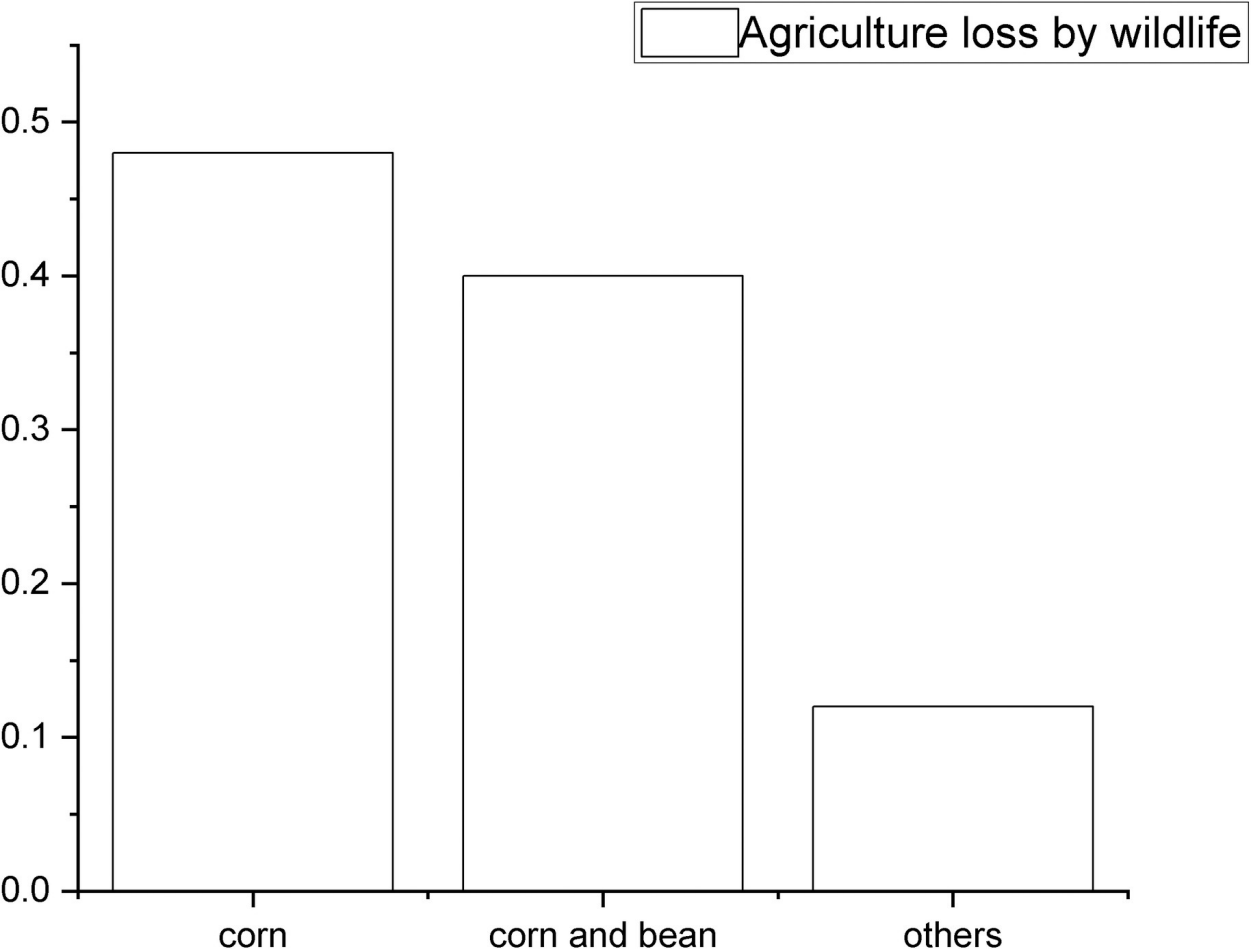
Sampling villages in the study area. Red squares indicate study villages. Two protected areas are shaded: the dark green region refers to the Land of Leopard National Park (Russia), and the light green region refers to Northeast China and Leopard National Park (China). Tiger occurrence was created based on previously published data [29] and base layers were created through ArcMap 10.3 (ESRI, Redlands, USA).

Our study area, Yanbian Korean Autonomous Prefecture, is located in the southeast of Jilin province, China, which borders both North Korea and Russia (Fig 2). Yanbian Korean Autonomous Prefecture contains 70% of Northeast China Tiger and Leopard National Park, a vast protected area [28].

The predominant economic activities in our study area are arable farming and livestock grazing, which also occur both in and around Northeast China Tiger and Leopard National Park. From May to October, cattle are grazed in the surrounding mountains; while from November to April, cattle are fed around human settlements with fodder.

### Questionnaire survey

We used stratified random sampling to select 27 villages in the research area to survey in the early spring (April to May) of 2017 using a structured interview questionnaire (S1 Table), which was administered in Chinese through face-to-face interviews [30].

Our study was approved by IRB (Institutional review board) of Seoul National University (No. 1608/001-011), participants were informed of the research purpose and verbal agreement to participate was obtained before starting the interview. Agreement form was signed the name by one witness at the first page of the questionnaire. A maximum of one adult member of each household was invited to participate in the interviews. All participants were over the age of 18 years old. Each survey was comprised of 48 questions, as well as several sub questions.

Survey questions were framed based on our understanding of the local communities and general wildlife issues in northeast China over more than ten years’ combined experience working in the region and with reference to the published literature [24, 31]. The questionnaire included questions about: a) basic demographic and socioeconomic information about respondents [32, 33]; b) respondents’ understanding of wildlife and its conservation [34–36], as well as their attitudes towards local wildlife [33]; and c) perspectives on human-wildlife conflict (for example, livestock predation, crop damage, or harm to humans) [36].

### Statistical analysis

We used descriptive statistics to analyse the demographic questions. We then conducted an exploratory factor analysis (EFA) to evaluate respondent attitudes towards wildlife and its conservation, which allowed us to explore potential relationships between responses [37]. In our survey questions 2.7.1–2.7.6, six target species were listed (S1 Table) and respondents were asked to indicate ‘preference’ towards a given species on a 5-point Likert scale, ranging from ’strongly dislike’’ to ‘like very much’ and with responses assigned a corresponding numerical value 1–5. We used 12 questions in the survey to measure perspectives towards wildlife, and five factors are reported in Table 3. These 12 attitude-related questions were selected for the EFA (original questions are reported in Part 2 of S1 Table). First, we conducted a Bartlett’s Test of Sphericity and a Kaiser-Meyer-Olkin (KMO) test for sample adequacy to determine whether the dataset could be analyzed using EFA [38, 39]. KMO can be used to test the adequacy of the sample size, with its values ranging from 0–1. If the value is lower than 0.6, it indicates that the dataset is not suitable for conducting the factor analysis. The KMO test returned a value of 0.72, which meant that our dataset was appropriate for analysis using an EFA [40]. Data was uploaded to IBM SPSS Statistics (Version 26), data reduction was selected through the analytical function and all variables were uploaded. In the context of a descriptive setting for the factor analysis, we selected the initial solution for statistics, together with KMO and Bartlett’s Test of Sphericity for the correlation matrix. In the rotation setting, we selected varimax method and rotated solution for the display. In the options setting, the suppress absolute values was set as 0.45. Other option settings were maintained as defaults.

For a clearer understanding of different groups, demographic variables including age, gender, and ethnicity were listed. In group one, the total number of participants was 70, that of group two was 40, and that of group three was 13 participants (S4 Table). Attitudes were scored from 1 to 5 on a Likert scale, presenting different levels of preference: strongly dislike (1), dislike (2), no feeling (3), like (4), like very much (5). After obtaining factors through the EFA, we applied the Hierarchical clustering method to set the number of groups for each factor and to check the demographic distribution of samples among the groups. A SPSS hierarchical cluster analysis was employed, the single solution number of clusters was set as 3, and the Euclidean distance measure was chosen; other settings were kept as default.

We used multiple linear regression to investigate which variables might influence the factors of attitude [16], and utilized the summed score of variables of each factors to represent each attitude factor index. Nine variables were selected as possible influencing factors of attitudes towards wildlife and wildlife conservation, including age, gender, education, annual income level, main source of income, personal experience of livestock depredation caused by wildlife, other agricultural loss to wildlife (e.g., arable crop loss), the number of cattle owned by the family, and whether a family were long-term residents or migrated to the region from somewhere else (typically, another region of China). We computed a multiple linear regression to investigate the possible relationship between variables and the attitude of local communities towards wildlife. All relevant analyses were conducted through IBM SPSS Statistics (Version 26).

## Results

### Respondent demographics

In our study, an annual income within a range of $2,000-$6,000 USD represented the largest proportion of households in our sample (38.76%). Government statistics show that in 2019, the Yanbian had an average GDP per capital of $ 5,000, less than half of the average GDP per capital of China ($ 10, 276) [27]. The main income sources in our study area were from corn and bean farming, cattle grazing, and part-time jobs (Table 1). Households with higher income levels tended to rely more heavily on cattle grazing and fungi farming, while those households with lower income levels depended more on arable farming. Individuals aged between 50 to 60 years old represented the largest proportion of respondents in our sample (32.3%). The most common education level was up to middle school (aged 13–16) (48.1%) (S2 Table). Compared with education statistics for the whole of Jilin province and China, the level of education to middle school in our research area was higher than that in Jilin or the national average for China, whereas further education levels (i.e. high school and tertiary education) were less than the provincial or national average [41] (S3 Table).

**Table 1:** Annual income and economy activities in the survey area. The ratio under each economy activity indicates the proportion of respondents who engage in these economic activities, with the majority of households involved in several different economic activities concurrently. The main income sources reported for the two lowest income tiers (annual income of $1,000-$2,500 and <$1,000 respectively) were from state support programs or financial support from other family members.

Income tier (n = 139)	Annual income	Economic activities			
		Arable farming (corn/bean)	Cattle rearing	Fungi harvesting	Part-time work
High-income (14.6%)	>$12,000	89.47%	26.31%	-	-
Middle-upper income (18.6%)	$5,000~$12,000	83.3%	41.47%	41.47%	-
Middle-income (38.76%)	$2,000~$6,000	89.8%	22.45%	-	28.58%
Middle-lower income (20.16%)	$1,000~$2,500	-	-	-	-
Low income (7.75%)	<$1,000	-	-	-	-

**Fig 3:**
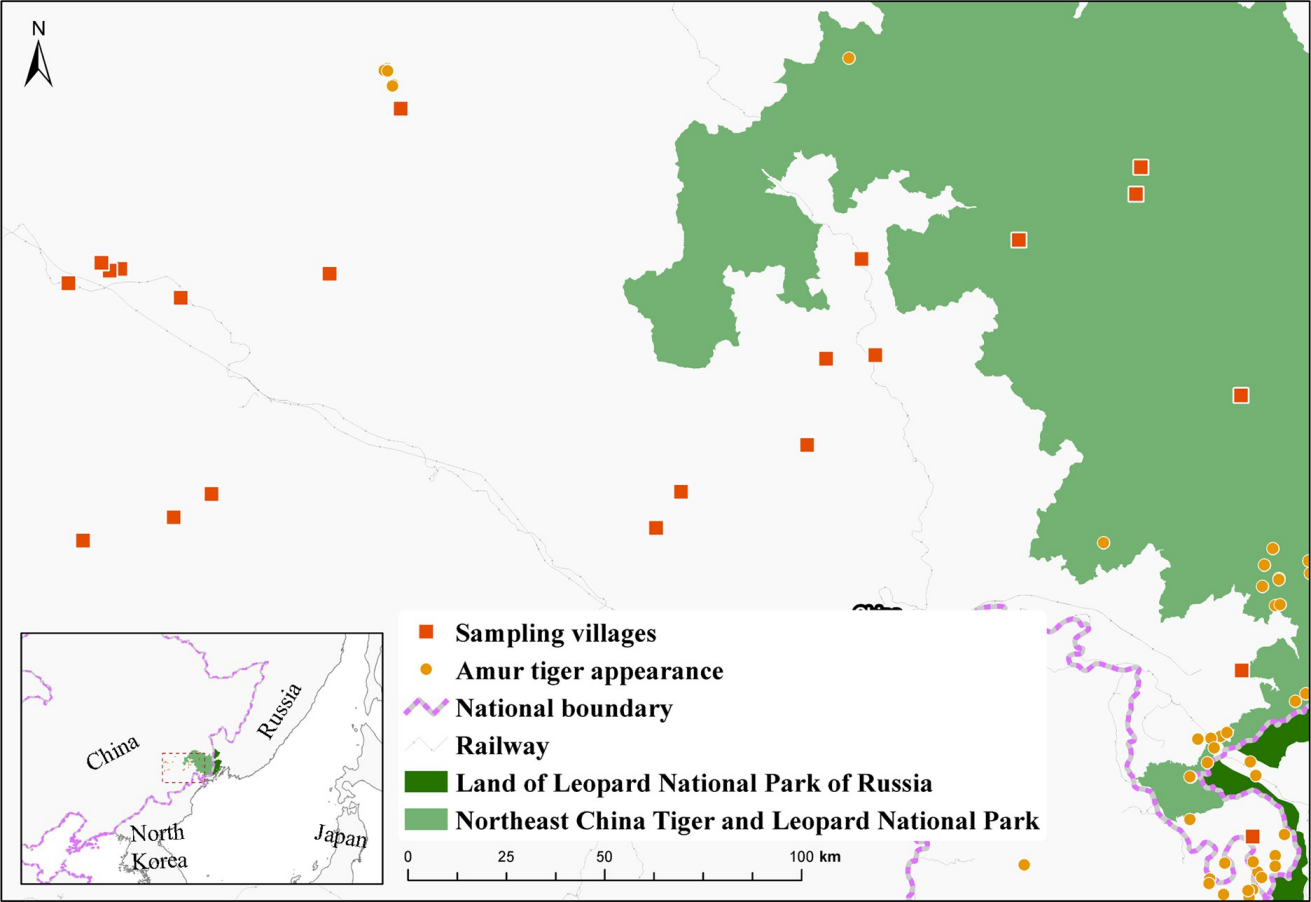
Agricultural product loss within 5 years. 48% of families reported that they had corn destroyed and 40% of families reported that they had lost both corn and beans to wildlife. Losses of other crops like ginseng and pumpkins were also reported.

When presented with images of the target species one at a time, over 90% of respondents knew and could identify tigers, leopards, and bears, but only half of the respondents could distinguish among different deer species. Five out of the 131 respondents reported having experienced of physical injuries from wildlife, of which two cases involving wild boar and three cases involving bears. No participant had experienced physical injury from big cats. 10% of the families surveyed had experienced loss of cattle or poultry to carnivores. Eight cases of livestock predation involved leopard cats (*Prionailurus bengalensis*), a small felid which typically predated poultry; one case involved predation by a bear; and there were three cases of cattle predation by tigers. Cattle owners received compensation from the government for livestock depredation caused by carnivores whereas other crops damaged by wildlife were not well compensated for in our study samples. 74% of families surveyed reported that they had lost an agricultural product due to wildlife within the last five years. Corn and beans made up a large proportion of the products lost to wildlife (Fig 3). In 24% of cases, losses were estimated to be more than half of the anticipated harvest. Among the 87 households that reported agricultural losses, the species involved were: wild boar (84 cases), roe deer (5 cases), bears (4 cases), badger (*Meles leucurus*) (2 cases), and pheasant (*Phasianus colchicus*) (2 cases).

### Attitudes towards wildlife

The total contribution of all factors is 72%. All the load coefficients are higher than 0.45 (Table 2), which means that there is a close correlation among all factors, and that common factors can be used to effectively explain the variables.

**Table 2:** Factor loading values based on exploratory factor analysis (N = 139). Total contribution of the factors is 72.5%, and all the loading coefficients are higher than 0.45, which means that there is a close correlation among each factor, and the common factors can be used to explain the variables effectively. By reading the loading values, 5 main attitude categories were classified as Factor 1, which could be described as the attitude towards carnivores; Factor 2 could be described as the preference towards herbivores; Factor 3 could be described as attitude towards reducing hunting to benefit wildlife conservation; Factor 4 could be described as the attitude towards wild boars; Factor 5 could be described as the attitude towards nature resources.

Factor content	Variable	Factor 1	Factor 2	Factor 3	Factor 4	Factor 5	Contribution
Attitudes towards large carnivores	B2. Attitudes towards tigers	0.895	-	-	-	-	21.4
	B8. Attitudes towards bears	0.859	-	-	-	-	-
	B3. Attitudes towards leopards	0.858	-	-	-	-	-
Attitudes towards herbivores	B6. Attitudes towards red deer	-	0.916	-	-	-	21.3
	B5. Attitudes towards sika deer	-	0.872	-	-	-	-
	B7. Attitudes towards roe deer	-	0.795	-	-	-	-
Attitudes towards a reduction in hunting to benefit wildlife conservation	B9. Agreement to the statement ‘the tiger population is small, so we should ban hunting’	-	-	0.799	-	-	10.6
	B12. Agreement to the statement ‘wildlife such as sika deer or roe deer do not negatively influence our life so we should not hunt them’	-	-	0.710	-	-	-
Attitude towards wild boar	B11. Agreement to the statement ‘the wild boar population is too big; we should set some snares in the mountains to hunt some’	-	-	-	0.746	-	10.0
	B1. Do you often watch wildlife documentaries?	-	-	-	0.519	-	-
	B4. Preference towards wild boar	-	-	-	0.467	-0.552	-
Attitude towards nature resource use	B10. Agreement to the statement ‘We have lived here for a long time and have the right to use natural resources’	-	-	-	-	0.872	9.2

(Extraction method principal component analysis; Rotation method: Kaiser normalized maximum variance method; Rotation converges after 7 interactions; KMO = 0.721,Bartlett’s Test of Sphericity = 0)

In our results, the ‘preference to tiger’, ‘preference to bears’, and ‘preference to leopards’ loading were 0.895, 0.859 and 0.858 respectively, and so factor 1 is described as attitudes towards large carnivores. Preference to red deer (*Cervus elaphus*), preference to sika deer (*Cervus nippon*) and preference to roe deer had loadings of 0.916, 0.872 and 0.795 respectively, which meant that these three variables have a strong correlation with factor 2, and the importance ranking is preference to red deer, sika deer and last is roe deer, so we can summarize the second factor as preference to ungulates; agreement to the statement, “the tiger population is small, so we should ban hunting” and agreement to the statement, “wildlife such as sika deer or roe deer do not negatively influence our life so we should not hunt them” had 0.799 and 0.710 loading values in factor 3, which can be summarized as preferences for reducing hunting to benefit wildlife conservation. Three variables are assessed in agreement to the statement that ‘the wild boar population is too big; we should set some snares in the mountain to hunt them’, the questions of ‘do you watch programs related to wildlife’, and the ‘preference to wild boar’ loaded 0.746, 0.519 and 0.467. In factor 4, the statement, ‘the wild boar population is too big, and we should reduce the population’ contributed the highest coefficient value which means it is the most important variable in factor 4, which could be summarized as the primary attitude towards wild boar. The agreement with the statement "we have lived here for a long time and have the right to use natural resources" was the only variable in the final factor, which had a loading value of 0.872. As a result, the final factor can be summed up as the dominant attitude toward the use of natural resources. Based upon their contribution, factor 1 has the highest contribution value, 21.357, factor 5 has the lowest contribution value, which is 9.165, and the total contribution is 72.5%, which means that the five factors can explain 72.5% of the total attitude characteristics we conducted (Table 2).

### Perceptions of tiger and other large carnivores

Cluster analysis was used to provide the attitude groups of the samples. Three groups showed the best performance among the results. Attitudes towards large carnivores including tiger, bears, and leopard were assessed. Group 2 had the highest mean value (4.15 for tiger, 4.1 for bear, 3.49 for leopard), which signifies that this group had the most positive perceptions of these species. Group 3 had the lowest mean scores (1.08 for tiger, 1 for bear, 1 for leopard), indicating a strong dislike of large carnivores. Most respondents were in group 1, whose score was between “like” and “no feeling”, (2.66 for tiger, 2.7 for bear, 2.53 for leopard). Group 1 and 2 included more respondents that liked predators and group 3 included more respondents who disliked them.

Together with attitude towards large carnivores, gender, ethnic group, and age, variables were grouped by using cluster analysis. It is possible to group the closed individuals who share a common attitude and comprehend their demographics characteristics. People in groups 1 and 2 were older than those in group 3. Group 3 included more females than groups 1 and 2. More Han-Chinese respondents were in group 1 and 2, while more respondents who came from ethnic minority groups were in group 3. The results of the clustering analysis showsthat elder respondents and Han-Chinese respondents were more likely to express opinions that indicated greater tolerance for large carnivores.

### Perceptions of prey species

There were more cases of interaction between humans and wild boar were reported than interactions between human and deer. The mean values of the response to the questions about the preference to sika deer, red deer and roe deer were 3.83, 3.69 and 3.41 respectively, which indicated a high level of positive attitudes towards each deer species. In the attitudes of the three groups, group 2 was comprised of 5.7% of the respondents who had a low score and disliked all deer species. Those in groups 1 and 3 had high scores for sika deer (3.36) and red deer (3.45), but comparatively low scores for roe deer (1.64).

A greater proportion of women than men expressed positive attitudes towards deer, while Han Chinese respondents showed more positive attitudes toward deer species than other ethnic groups did (S5 Table). Attitudes towards deer did not vary by age throughout the groups. By contrast, the average score of preference to wild boar was 1.75, between strongly dislike and do not like. Just 4.1% of individuals expressed that they ‘like’ this species. 44.3% of individuals agreed that the wild boar population was too big and needed to be reduced, for example, by using snares. More tolerant attitudes towards wild boar were more likely to be expressed by male respondents, as well as older respondents (S6 Table).

### Variables influencing attitudes towards wildlife

We found that annual income level was negatively correlated with positive attitudes (‘like’, ‘like very much’) towards large carnivores (coefficient = -0.217; p<0.05). Participants who had migrated to Yanbian from outside the study area typically expressed lower tolerance towards large carnivores (coefficient = 0.192; p<0.05) and different deer species (coefficient = 0.220; p<0.05). Families with more income sources were more likely to express negative attitudes towards conservation (coefficient = -0.223; p<0.05) and those participants who had experienced loss of agricultural products showed a lower preference to wild boar and a high preference for the control of the wild boar population (coefficient = -0.299; p<0.01). Higher income respondents (coefficient = 0.231; p<0.05) and those who had experienced livestock depredation by wildlife (coefficient = 0.181; p<0.05) were more likely to express stronger views in favor of being able to use natural resources freely (Table 3).

**Table 3:** Important variables associated with the attitude towards wildlife using multiple linear regressions (n = 120). A regression model with nine potential influencing variables was run for each of the five attitude factors to determine their values. The F value and variable numbers were taken into consideration when selecting the result. Results in bold font represent highly significant values.

Variables	Attitudes towards large carnivores		Attitudes towards deer species		Attitudes towards conservation		Attitudes towards wild boar		Attitudes towards free use of natural resources	
	*Std*. *Beta coefficient*	*Sig*.	*Std*. *Beta coefficient*	*Sig*.	*Std*. *Beta coefficient*	*Sig*.	*Std*. *Beta coefficient*	*Sig*.	*Std*. *Beta coefficient*	*Sig*.
Age(A1)	0.185	0.069	0.12 3	0.230	0.064	0.507			-0.114	0.246
Gender(A2) *	-	-	0.107	0.239					0.066	0.459
Education level(A5)	0.180	0.064	-0.066	0.508	0.097	0.317	-0.127	0.184		
Annual income level(A8)	**-0.217**	**0.030**	-0.127	0.208			0.108	0.273	**0.231**	**0.022**
Main source of income (A10)	-	-	**-**	**-**	**-0.223**	**0.014**	-0.129	0.164	-0.049	0.586
Whether any livestock have been lost to wildlife (A14) **	-	-	**-**	**-**	-0.126	0.163	0.053	0.552	**0.181**	**0.044**
Whether any crops have been lost to wildlife (A15) **	-	-	**-**	**-**	**-**	**-**	**-0.299**	**0.002**	**-**	**-**
Number of cattle owned (A16)	-	-	**-**	**-**	**-**	**-**	**-**	**-**	**-**	**-**
Whether migrated to the region from another area (A17) ***	**0.192**	**0.035**	**0.2203**	**0.029**	**-**	**-**	0.046	0.618	**-**	**-**
*F-statistic*	3.598		2.326		2.595		2.374		3.051	
*Sig*.	0.008		0.047		0.040		0.034		0.013	
*Adjusted R* ^*2*^	0.079		0.052		0.050		0.064		0.079	
*The regression equation’s standard error of the estimate*	2.846		2.598		1.165		1.929		1.064	

*Male = 1, Female = 2

** Yes = 1, No = 0

*** Yes = 1, No = 2

## Discussion

Respondents generally expressed fairly neutral attitudes towards each of the large carnivores (S4 Table, 90% of respondents were close to ‘neutral’), including Amur tigers, Amur leopards, and bears, but showed a strongly negative attitudes towards wild boar (96% of the respondents were between ‘dislike’ and ‘strong dislike’, S6 Table). Respondents expressed generally mildly positive attitudes towards deer species (between ‘neutral’ and ‘like’), including roe deer, sika deer and red deer (S5 Table). Our results are encouraging for large carnivore conservation in northeast China given that in many examples around the world, large carnivores are perceived extremely negatively by the communities they live alongside, due to their potential threat to human life and property [42, 43]. This may be encouraged by the observation in our results that there were no reported cases of human casualties, while loss of livestock to large carnivores appears to currently be relatively unusual and where the loss of livestock to tigers does occur, farmers receive compensation from the government-led compensation program. However, even though the probability is currently very low, there could be increasing risks to human life or property as the population size of tigers in the region increases in the future, as seen in some cases in India [44]. Even a few cases of injury to humans by large carnivores may contribute significantly increasing negative attitudes towards large carnivores. Thus, for long-term conservation strategies for large carnivores to be successful, in addition to programs aiming at effective carnivore-human conflict prevention and mitigation, detailed plans to improve local community’s attitudes towards large carnivores need to be formulated and included in the strategies. For example, developing and implementing school-based conservation education programs targeted at children and students might be an effective strategy to influence their parents’ attitude towards large carnivores, as has been shown for jaguar (*Panthera onca*) conservation in the Brazilian Amazon [45]. At the same time, it may be appropriate to regulate and limit human activities near the national park in order to reduce the potential for conflict with large carnivores [46].

The strongly negative attitude expressed towards wild boar is a concern for tiger conservation because the species is one of the most important prey animals for Amur tigers; thus, negative attitudes towards wild boar could also have a negative impact in the long-term for tiger conservation. We suspect that the strongly negative attitude towards wild boar is likely generated by heavy crop damage caused by wild boars in the region (Table 3). Thus, reducing crop damage caused by wild boars may contribute to the improvement of local peoples’ attitude towards them in particular and also towards wildlife in general. Large carnivore conservation education programs might utilize these findings of local community’s relatively neutral attitude towards large carnivores and negative attitudes towards wild boars. For example, conservation education programs may wish to incorporate the fact that conserving healthy large carnivore populations may reduce crop damage by reducing the density of wild boar population [47]. Reduction of crop damage by wild boars may eventually contribute to the reduction of the local community’s negative attitude towards wild boars.

### Targeting groups and conservation solutions

The support of residents plays an important role in the sustainable conservation of wildlife [18]. Understanding residents’ attitude status is an important first step in ensuring that conservation strategies have the support of residents. In our results, greater proportions of negative attitudes were expressed among younger respondents and respondents from ethnic minority groups (S4 Table). Respondents who had moved from other areas of China showed less tolerance towards target wildlife compared with long-term residents (Table 3). Higher income families held more negative attitudes towards large carnivores and expressed a stronger willingness to freely use natural resources (Table 3). Families that had experienced cattle depredation also held a stronger willingness to use natural resources freely and those who had experienced crop damage caused by wildlife were more likely to hold negative attitudes towards wild boar. Surprisingly, the more income sources a family had, the less likely they were to support wildlife conservation. This might be caused by the fact that families with more sources of income were more likely to rely on different natural resources.

The support of residents plays an important role in the sustainable protection of wildlife [18]. Understanding residents’ attitude status is the important first step for ensuring that conservation strategies have the support of residents. When a new protected area is established, the introduction of conservation measures such as hunting controls, access limitation, or other limitations on resource use are often implemented, which may prompt the development of negative attitudes from the local community towards both the regulations and species that are primarily targeted for protection [48].

Targeted conservation messaging will likely be important for the success of local conservation initiatives. Our results indicated that ethnic minority groups showed less positive attitudes towards large carnivore species (S4 Table) and high-income level families were more likely to have negative attitudes (Table 3). We initially assumed that because of their unique culture, the Korean Chinese ethnic minority, which has many folktales and stories related to wildlife, particularly tigers [49], might have some positive influence on their attitude toward wildlife. However, the opposite result was found, which may be due to their similar lifestyle to other ethnic groups in this region for a long time existing old culture or stories may no longer be relevant. During interviews, we collected information on what participants did in their free time, in order to ascertain how conservation initiatives could best target local residents. Watching TV was the most frequently given answer, with the popular channels being CCTV (Chinese Central Television Channel) channels 1, 2, 4, 13 and Jilin provincial channels. We suggest that these might be suitable outlets to provide environmental education programming to support the objectives of wildlife conservation. High-income families, who were more likely to have negative attitudes towards target wildlife (Table 3), and migrants from other regions of China might need a tailored engagement strategy; for example, introducing an economic strategy for wildlife-friendly products as an alternative livelihood source [50].

### Compensation for livestock depredation

Our results, as well as those of other studies, have shown that livestock depredation could contributed to negative attitudes towards large carnivores [51–54], The current compensation strategy employed may help, but not sufficiently mitigate, negative attitudes resulting from human-wildlife conflict. In our study, high-income level groups were more likely to hold negative attitudes towards carnivores and one of their main economic activities was from livestock grazing (Table 3). Families who had experienced livestock depredation expressed a stronger willingness to freely use natural resource freely (Table 3), which might result in grazing in protected areas. In Yanbian, communities maintain a traditional way of cattle grazing, which involves leaving cattle to graze freely in the mountains during the summer months and cattle owners will only occasionally feed them with salt. Cattle grazing in the forests occurred from May to October, therefore, compared to winter and early spring when cattle were fed inside, there was a higher risk of carnivore predation of livestock in the summer. Traditional cattle grazing practices existed long before Jilin Hunchun Amur tiger nature reserve was established.

Financial compensation for losses caused by human-wildlife conflict is an important conservation tool that has been used in a wide range of circumstances around the world [25, 55]. Jilin province started compensating for losses caused by wildlife in 2006, under the program 吉林省重点保护陆生野生动物造成人身财产损害补偿办法 *(English*: *‘key protected terrestrial wildlife property damage compensation regulation’)* [56]. Until 2021, based on wildlife protection laws in China, 9 provinces, including Jilin, formulated consistent compensation regulations. The main focus of the regulations was to compensate the losses caused by wildlife, rather than reducing human-wildlife conflict [24]. By contrast, in some cases such as in southern Kenya, livestock is required to be kept in a predator-proof enclosure every night [57], while in Greece, appropriate preventative measures would need to be taken if the depredation occurred repeatedly in the same location [58].

For cattle owners, the economic loss of cattle depredation caused by large carnivores was low after the implementation of the compensation program. High-biodiversity regions are often in areas with low levels of economic development [59, 60]. In these circumstances, economic losses caused by wildlife can generate negative attitudes towards wildlife and their conservation [24, 61]. By 2009, $1.29 million (USD) had been used for such compensation in Jilin, among which, 8.96% involved tigers, with other cases involving bears or migratory birds [24]. Although it is prohibited to graze livestock within the protected area, compensation is still paid even if livestock is predated inside the nature reserve’s boundaries (Jilin Hunchun Northeast Tiger Nature Reserve), which was established in 2001. In 2017, Hunchun Forestry Department records show that 180 cattle and 3 horses had been predated by tigers and just 5 owners did not get compensation because the evidence of tiger depredation was not clear. The high percentage of awarded compensation may inadvertently encourage livestock grazing in protected tiger habitat. Grazing in the forest may also be an important threat to other wildlife populations because of the spread of diseases or competition for grazing with other herbivores [62–64]. When considering how to sensitively reduce the practice of grazing cattle in forest areas, cooperation with cattle owners will be important. Given that this may be an example where compensation has unintended negative consequences [65] and there are additional risks associated with cattle grazing in protected forest areas, we recommend gradually removing the payment of compensation in circumstances where depredation occurs within protected areas, so as to encourage local communities to avoid grazing in core habitat for tigers and other large carnivores.

### Wild boar management

Our results showed that 95% of respondents expressed negative attitudes towards wild boar (S6 Table). The main loss of agricultural products was corn, which was also found in Spain [66]. Conservation of the wild boar population in our study area is important for tiger conservation, as wild boar is an important prey species for the Amur tiger and the species plays an important role in the composition of the ecosystem’s large mammal biomass [67]. Previous research showed agricultural landscape features that have been linked to wild boar damage, for example Amici et al. (2011) highlighted the likelihood of a high density of wild boar damage in areas close to forests and rivers [68], as in our study area. Predicting where crop damage caused by wild boar is likely to occur would be useful for preventing human-wild boar conflict [69]. From our survey, we also found that less than half of the families surveyed utilized practices for preventing wild boar damage to farmland (47 in 140). The main practice was using firecrackers or other tools to make noises, in order to scare off wild boar (20 out of 47 cases), and half of respondents thought that this practice was effective. Other practices used by the local community included building plastic fences (a simple fence made by used plastic sheet) or lighting fires. We point out that it would be incredibly unlikely to completely eradicate crop raiding brought on by wild boar, but it would be possible to lessen the damage caused by wild boar by building electric fences [70, 71], although doing so would result in an increase in the amount of local electricity used. Smearing an oil mixture made of domestic pig fat around arable land has been shown to potentially reduce crop raiding damage by 60–80% [72], and burning dried pig dung has been shown in other studies to reduce crop damage by 35%–50% [72]. These are just two examples of traditional management approaches that have been demonstrated to work well in practice in India. In areas with large wild boar populations, additional measures, such as cultivating alternative agriculture crops, may also be helpful [66].

Conservation activities which might provide benefits to communities can be as useful tools to gain the support for the wildlife conservation [50]. Such programs would ideally involve local community members (including those who have experienced crop damage by wild boar or other herbivores, or loss of livestock to carnivores), conservation groups, researchers as well as local authorities, who will necessarily help mitigate conflicts and contribute to positive attitudes towards wildlife. In addition to wild boar, roe deer are likely to play a significant role in determining how the local community views wildlife. (S5 Table). In the clustering result of attitudes towards deer species, local communities expressed less preference towards roe deer compared with other deer species. Crops in agricultural lands were confirmed as one of the most important components of the diets of European roe deer (*Capreolus capreolus)* in France, Poland, The Czech Republic, and Hungary [73] and, more recently, as important habitat for Asian (Siberian) roe deer [74]. Compared to other deer species, roe deer make extensive use of small woodlands and agricultural lands [75]. Crop damage may influence the attitudes of the community towards roe deer, as in the case of wild boar (Table 3). Conflicts between tiger prey species and human communities could lead farmers to take direct or indirect revenge on those ungulate species, which could negatively influence tiger conservation in the long term [76].

### Study limitations

While this study provides valuable insight into community perceptions of endangered large carnivores and their prey species in an understudied region, it has some important limitations. While Yanbian is an area of national importance for tiger conservation in China, the density of tigers and other large carnivores varied in different areas of the region. We used random sampling but were not able to collect enough samples to reliably compare the difference in attitudes among communities living in areas of the region with comparatively higher and lower densities of tigers. In the linear regression analysis, more observations would be contributed by incorporating village difference and applying a random effect model, but in our sampling, not enough samples in different areas were collected, so only one fixed effect was used to consider one group as explanatory variable. Another limitation is that while our results showed that families who had migrated to the region might express less tolerance towards target wildlife, we did not record data on how long individual families had lived in the region or where in China they had migrated from, which might provide further insight into this finding (for example, whether their perceptions changed over time). Furthermore, other factors, such as policy implementation of conservation actions, might also potentially influence community attitudes.

## Conclusion

Our study shows that local communities in our study area of Yanbian Korean Autonomous Prefecture in northeast China, an area of national importance for tiger conservation, generally hold relatively neutral attitudes towards large carnivores, but showed strongly negative attitudes towards wild boar, likely due to crop raiding. The development of more targeted and nuanced conservation education programs, tailored respectively for high income families; residents who are recent migrants from other regions of China; and local ethnic minority groups, may be needed to ensure widespread societal support for large carnivore conservation and the conditions essential for its success (such as a sustainable ungulate population). Monetary support, in the form of compensation for damage resulting from big cats, is clearly a valuable tool for successfully encouraging positive attitudes towards big cats and big cat conservation in rural communities, particularly where there are high levels of poverty and subsistence farming. However, we suggest that the system of compensation currently used can be improved: in order to reduce livestock grazing in protected areas which are intended to serve as tiger habitat, with the aim of reducing the potential risk of human-tiger conflict, it would be beneficial to have a detailed compensation plan where no compensation is provided for the loss of livestock within the core protected areas. Different groups may also need different types of support. For example, when the livelihoods of household livelihoods have been directly impacted by wildlife (e.g., the loss of agricultural products to ungulates, which are important tiger prey species), financial compensation may be appropriate, as would the trial and introduction of techniques to reduce crop-raiding by wildlife.

## Supporting information

S1 Table Questionnaire sheet. (DOCX)

S2 Table Demography information. (DOCX)

S3 Table Education status in China and Jilin. (DOCX)

S4 Table Demography information in different groups of attitudes towards large carnivores. (DOCX)

S5 Table Demography information in different groups of attitudes towards deer species. (DOCX)

S6 Table Demography information in different groups of attitudes towards wild boars. (DOCX)
