# Supplementary material for: Community attitudes towards Amur tigers (Panthera tigris altaica) and their prey species in Yanbian, Jilin province, a region of northeast China where tigers are returning
Source: PLoS One. 2022 Oct 27;17(10):e0276554. doi: 10.1371/journal.pone.0276554 (PMC9612539; doi:10.1371/journal.pone.0276554)
Supplement: S1 Table — (DOCX) [file pone.0276554.s001.docx]

| Researcher ： Contact (telephone/WeChat/QQ）：  Research date：  Region：1. Yanji 2. Tumen 3. Dunhua 4. Hunchun 5. Longjing 6. Helong 7. Wangqing 8. Antu  9. Shulan 10. Wuchang 11. Ningan 12. Dongning 13 Other Religions： |
| --- |

**S1 Table Questionnaire Sheet.**

| **Region name**  （e.g.：Name of village committee; Name of forestry farm） | |
| --- | --- |
| **Note** (overall feeling after survey) | 1）Very plesant communication 2）question-answer 3）not very cooperative 4）refused 5）other feeling or information： |

**1.Basic Information**

- 1. **Age** 1. 18-20 2. 20-30 3. 30-40 4. 40-50 5. 50-60 6. >60

**1.2. Gender** 1. Male 2. Female

**1.3. Household population:**

**1.4. Ethnic group**  1. Chinese 2. Korean Chinese 3. Manchu 4. Hui 5. Other

**1.5. Religious belief** 1. Christion 2. Buddhism 3. Shamanism 4. Other： 5. None

**1.6. Education level**  1. None 2. Under primary school 3. Primary school 4. Middle school 5. Technical secondary school 6. High school 7. College 8. University 9 Graduate school

**1.7. Where were you born？**

1. Another place. Why move here? 2. Local

**1.8. Are there more or fewer local residents than before?**

1 More. What is the reason do you think?

2 Fewer. What is the reason do you think?

3 No change

**1.9. Will your children live here in the future?**  1 Yes 2 No 3 Not sure 4 No children

**1.10. Education of your children？**1.None 2. under primary school 3. Primary school 4. Middle school 5. Vocational High School 6. High school 7. College 8. University 9 Graduate school

- 1. **Your vies on rural children receiving higher education?**

1.Strongly support. It is very important, and I will try everything to support children for school.

2.Support and will afford their education within the ability

3.Neither support nor oppose

4.Against. Going to college may not lead to a good job. It’s useless.

5.Strongly against. I will not allow children go to school and will persuade others to do so.

**1.12. what do you think is the main development industry in your area?** 1. Agriculture 2. Forest 3 Tourism 4 Industry (like factory) 5 Estate 6 Other

## 1.13. What’s your family’s annually income?

1.Less than 5000

2.5000~15.000

3.15.000~30.000

4. 35.000~80.000

5. 80.000~10.000

6. more than 10.000

**1.14. Are you satisfied with your life？** 1. Very satisfied 2. Normal 3.No

If no, what is the reason?

1. Low Income
2. Medication
3. Transportation
4. Human relationship
5. Hygiene issue
6. Education
7. Other

## ※※1.15. What are your family’s main activities? (Investigators need to be patient and cross examine one by one)

| **Category** | **content** | **（if yes√, and write the scale）** | **Annually profits（unit:10.000）** | **Category** | **content** | **（if yes√, and write the scale）** | **Annually profits（unit:10.000）** |
| --- | --- | --- | --- | --- | --- | --- | --- |
| 1.Farming（囗close to forest；囗inside forest；囗far away from forest） | Bean | 囗（ ） |  | 7. Grazing | Cattle | 囗（ ） |  |
|  | Corn | 囗（ ） |  |  | Sheep | 囗（ ） |  |
|  |  |  |  |  | Dog（囗Rabies Vaccine） | 囗（ ） |  |
| 2.Rice | Rice | 囗（ ） |  | 8.Forestry economy products | NTFP | 囗 |  |
| 3.Economic products | Ginseng | 囗（ ） |  |  | Firewood | 囗 |  |
|  | Medicine | 囗（ ） |  |  | Frog | 囗 |  |
| 4.Shop |  | 囗 |  | 9.Parttime job |  | 囗 |  |
| 5.Forest occupation compensation |  | 囗（ ） |  | 10 Fungus farming |  | 囗（ ） |  |
| 6.Land occupation compensation |  | 囗（ ） |  | 11. Workers |  |  |  |
| Other2） |  |  |  | Other3） |  |  |  |

**1.16. What do you think is the main source of income for the family：**

1. Farming
2. Grazing
3. Payment jobs
4. Shop
5. Forestry economy
6. Other

## 1.17. What is your entertainment when you are free：

1. TV
2. Travel
3. Internet entertainment
4. Exercise
5. Reading
6. Movie
7. Other

| 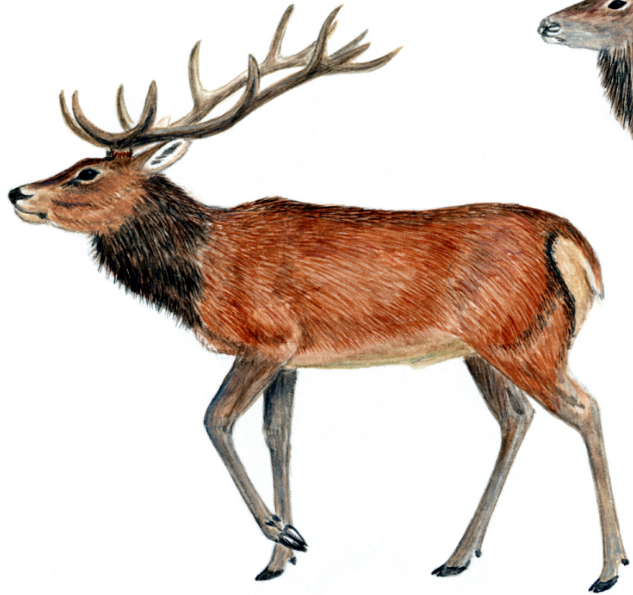 |
| --- |
| 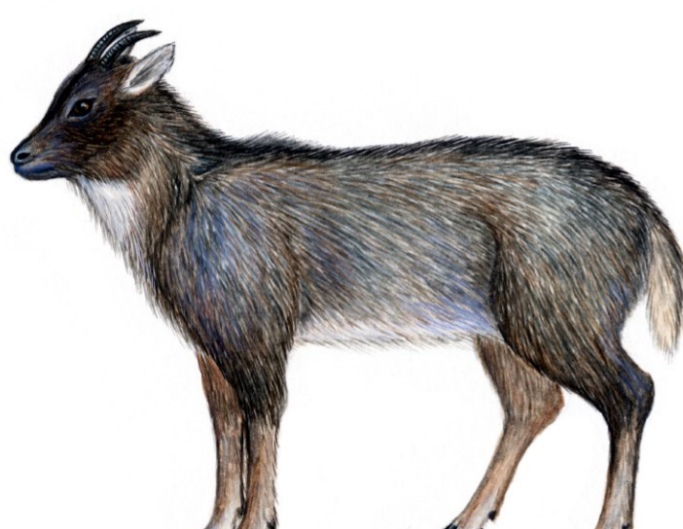 |
| 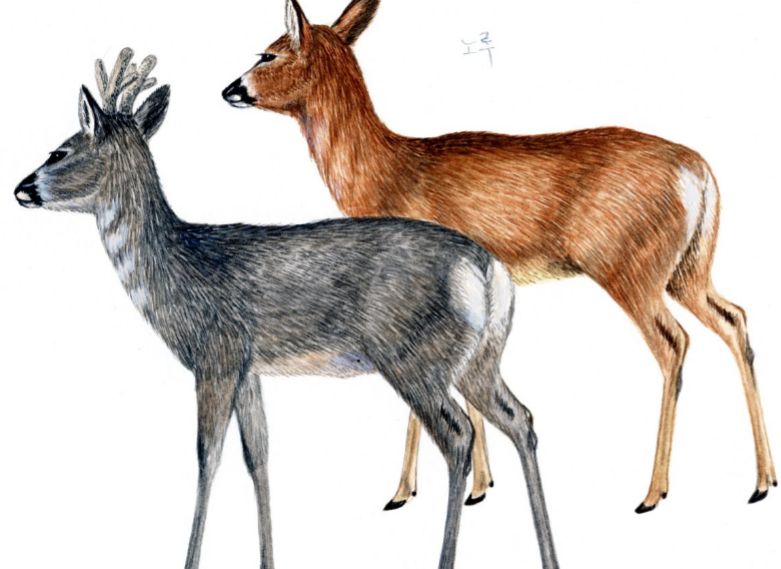 |
| 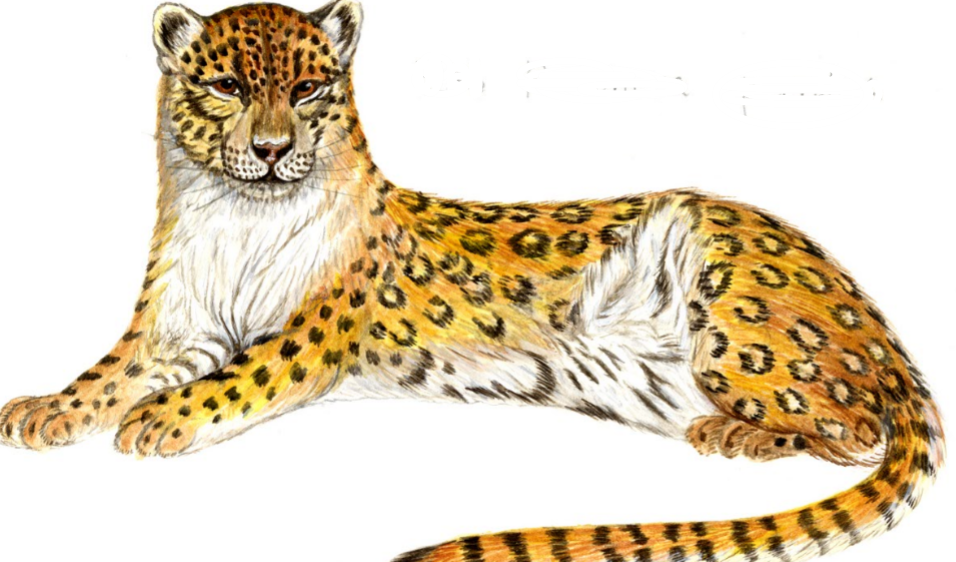 |
| 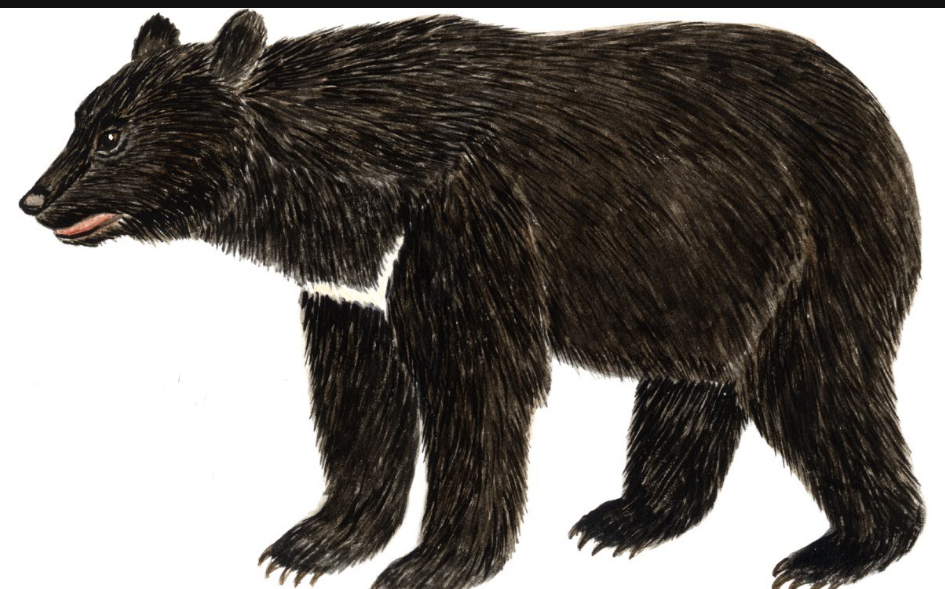 |
| 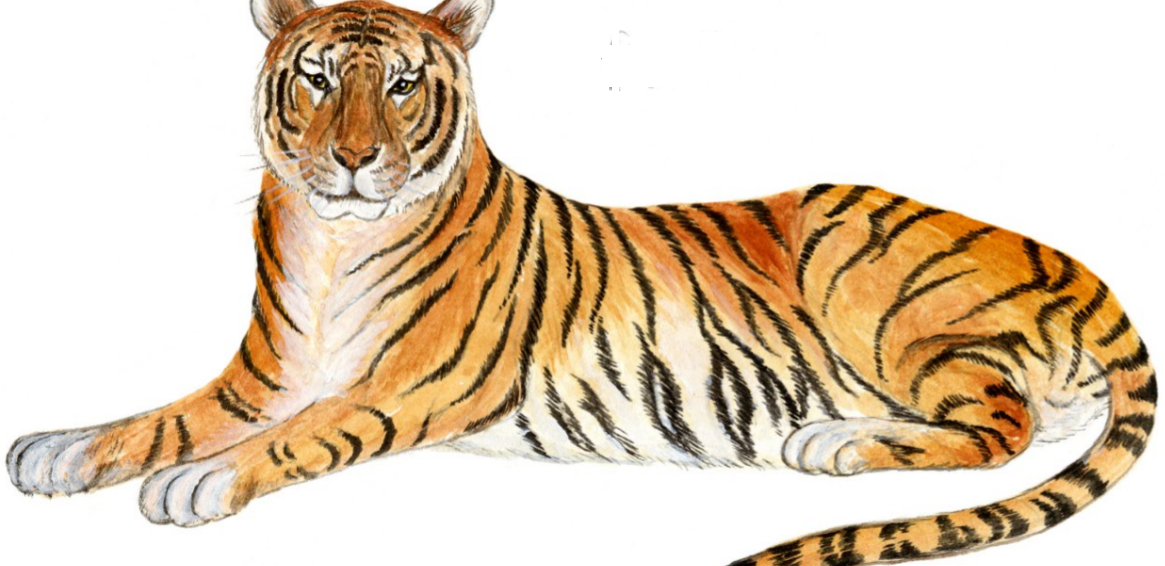 |

# Attitudes towards wildlife and conservation

**2.1. Do you know the following animals? If yes, tick, and fill in th names**

□ 2.1.1

□ 2.1.2

□ 2.1.3

□ 2.1.4

□ 2.1.5

□ 2.1.6

**2.2. What kind of wild animals are there in the local area（you have seen and heard of them, multiply choices/）** 1. Tiger 2. Leopard 3. Lynx 4. Sika deer 5. Wild boar 6.Roe deer 7.Bear 8. Red deer 9. Fox 10. Badger 11. Wolf 12. Other 13. Not clear

**2.3. Do you usually watch TV programs like *Animal Wor*ld and *Exploring Nature*?** 1. Not really 2. Once or twice a year 3. Over 3 times a year

**2.4. When did you first hear "wildlife conservation"？** 1. 20 years before 2. 10 years before 3. Less than 10 years 4. Less than 5 years 5. Didn’t heard about it

**2.5. Where do you get those information normally？**

1. Government 2. TV 3. Video 4. Community 5. Book and magazine 6. Known people 7. University students 8. Internet

**2.6. Is there any change after the wildlife conservation?**

1. No

2. Yes in what ways?

3. Have loss

**2.7. Please select your preference or hate level to the wildlife**

|  | **1.Strongly dislike** | **2.Dislike** | **3.Neutral** | **4.Like** | **5.Like them very much** |
| --- | --- | --- | --- | --- | --- |
| **2.7.1. Tiger** | 1 | 2 | 3 | 4 | 5 |
| **2.7.2. Leopard** | 1 | 2 | 3 | 4 | 5 |
| **2.7.3. Wild boar** | 1 | 2 | 3 | 4 | 5 |
| **2.7.4. Sika deer** | 1 | 2 | 3 | 4 | 5 |
| **2.7.5. Red deer** | 1 | 2 | 3 | 4 | 5 |
| **2.7.6. Roe deer** | 1 | 2 | 3 | 4 | 5 |
| **2.7.7. Bear** | 1 | 2 | 3 | 4 | 5 |

**2.8. Please circle the number that best fits your attitude**

1. Strongly disagree
2. Disagree
3. Neutral
4. Agree
5. Strongly agree

2.8.1. There are not too many tigers, so we need to protect them.................................................1 2 3 4 5

2.8.2. ‘We live here for long time, so we cannot use resources freedom’ such statement is wrong …...................1 2 3 4 5

2.8.3. ‘There are too many wild boars kill some will be fine’ such statement is wrong. .............................1 2 3 4 5

2.8.4. Animals like deer are not harming us so we agree the idea to conserve them ………………….1 2 3 4 5

2.8.5. If outsiders come to our place to hunt, we are not agree…....................................1 2 3 4 5

2.8.6. Leopard conservation is good for us…………….................................................................1 2 3 4 5

2.8.7. Tigers will not harm livestock.............................................................................................1 2 3 4 5

2.8.8. Tigers do not eat human beings………....................................................................1 2 3 4 5

2.8.9. If we encounter wildlife related problems, we can easily contact the staff of wildlife protection agencies

........................................................................................................................................1 2 3 4 5

## 2.9. If the wildlife harm our farmland can we set snares to prevent？1Yes 2 No 3 Do’t know 4. Have not done it before

2.10.　What changes do you think have taken place in the frequency of livestock being preyed on by wild animals in recent years？ 1. Increase 2. Decrease 3. No change 4. Not clear

2.11. Do you think there has been any change in the frequency of crops being harmed by wild animals in recent years？1. Increase 2. Decrease 3. No change 4. Not clear

**3. Human-wildlife conflict**

**3.1. Do you or your family have the experience of being attacked by wildlife？**1. Yes 2. No

**3.1.1. If yes what wildlife？**1. Tiger or leopard 2. Bear 3. Wild boar 4. Deer 5. Other

**3.1.2. Injury status** 1. Dead 2. Serious damaged 3. Not serious injured 4. Scared 5. Other

**3.1.3 Time**

**3.1.4. Did you get the compensation** 1. Yes （If yes, do you satisfied?：1. Yes 2. No） 2. No

Questions will talk with the family who has livestock. If they do not have any please jump to next question

**3.2. Was an livestock attacked by wildlife in your family？** 1Yes 2No

**3.2.1. If yes, by what kind of wildlife？**1. Tiger or leopard 2. Bear 3. Wild boar 4. Deer 5. Other

**3.2.2. Livestock that got hurt.** 1. Cattle 2. Sheep 3. Horse 4. Dog 5. Chicken 6. Other

**3.2.3. Injury status** 1. dead 2. Serious damaged 3. Not serious injured 4. Scared 5. Other

**3.2.4. Time**

**3.2.5. Did you get the compensation**  1. Yes （If yes, do you satisfied?：1.Yes 2. No） 2. No

**3.2.6. Frequent seasons:** 1. Spring 2. Summer 3. Autumn 4. Winter

3 损失程度对家庭的影响 1. 非常严重 2. 不太严重 3. 不严重

4 时间

1. 是否得到补偿 1. 是 （如果是，补偿是否满意：1. 满意 2. 不满意） 2. 否
2. 经常发生的季节：1. 春 2. 夏 3. 秋 4. 冬

**3.3. Did you hire someone look after your livestock?**  1. Yes 2. No

- If not, how often do you go to check them?

**3.4. Do you have any plan to prevent the loss on livestock？** 1. Yes 2. No

If yes, what is it? Is that effective 1. Very effective 2. Not so much 3. No

**The following questions 3.5-3.6 are for families with farmland. If there is no farmland, skip to next topic.**

**3.5. Have any wild animals ever damaged your crops？**  1. Yes 2. No

**3.5.1. If yes what wildlife？**1. Tiger or leopard 2. Bear 3. Wild boar 4. Deer 5. Other

**3.5.2.Type of crops:** 1. Bean 2. Corn 3. Horney 4. Others

**3.5.3. Amount of loss:** 1). Less than the total product 1/5 2). Around 1/3 3). More than half

**3.5.4.Time**

**3.5.5. Did you get the compensation** 1. Yes （If yes, do you satisfied?：1. Yes 2. No） 2. No

**3.5.6.The most frequent time:** 1. Spring 2. Summer 3. Autumn 4. Winter

**3.6. Did you do anything to prevent the loss？** 1. Yes 2. No

If yes

**3.6.1. What is it?**

**3.6.2.Is it effective？**1. Very much 2. So so 3. Not effective

**Thank you so much for your support!**
