# Supplementary material for: Community attitudes towards Amur tigers (Panthera tigris altaica) and their prey species in Yanbian, Jilin province, a region of northeast China where tigers are returning
Source: PLoS One. 2022 Oct 27;17(10):e0276554. doi: 10.1371/journal.pone.0276554 (PMC9612539; doi:10.1371/journal.pone.0276554)
Supplement: S2 Table — (DOCX) [file pone.0276554.s002.docx]

**S2 Table. Demography information**

| **Variable** | **Description** | **Count** | **Effective percent (%)** |
| --- | --- | --- | --- |
| Gender | Female | 59 | 44.7 |
|  | Male | 73 | 55.3 |
| Age | <20 | 1 | 8 |
|  | 20-30 | 10 | 7.7 |
|  | 30-40 | 12 | 9.2 |
|  | 40-50 | 30 | 23.1 |
|  | 50-60 | 42 | 32.3 |
|  | >60 | 35 | 26.9 |
| Education | No education | 8 | 6.2 |
|  | Dropped out from elementary school | 5 | 3.9 |
|  | Graduate from primary school | 30 | 23.3 |
|  | Graduate from middle school | 62 | 48.1 |
|  | Graduate from vocational high school | 2 | 1.6 |
|  | Graduate from high school | 14 | 10.9 |
|  | Graduate from Junior college | 5 | 3.9 |
|  | undergraduate | 2 | 1.6 |
|  | Postgraduate | 1 | 0.8 |
