# Supplementary material for: Community attitudes towards Amur tigers (Panthera tigris altaica) and their prey species in Yanbian, Jilin province, a region of northeast China where tigers are returning
Source: PLoS One. 2022 Oct 27;17(10):e0276554. doi: 10.1371/journal.pone.0276554 (PMC9612539; doi:10.1371/journal.pone.0276554)
Supplement: S3 Table — (DOCX) [file pone.0276554.s003.docx]

**S3 Table. Education status in China and Jilin**. The 2020 Chinese government research of education in China (Ning, 2021).

| **Region** | **Primary school education** | **Middle school education** | **High school education** | **Higher level above high school** |
| --- | --- | --- | --- | --- |
| China | 24.77% | 34.51% | 15.01% | 15.47% |
| Jilin province | 22.32% | 38.23% | 17.08% | 16.74% |

Ning, J. 2021. Main data information of the seventh National Census. In: N. B. o. S. o. China (ed.).
