# Supplementary material for: Community attitudes towards Amur tigers (Panthera tigris altaica) and their prey species in Yanbian, Jilin province, a region of northeast China where tigers are returning
Source: PLoS One. 2022 Oct 27;17(10):e0276554. doi: 10.1371/journal.pone.0276554 (PMC9612539; doi:10.1371/journal.pone.0276554)
Supplement: S4 Table — (DOCX) [file pone.0276554.s004.docx]

**S4 Table. Demography information in different groups of attitudes towards large carnivores.** In the variables, age value from 1=18-20, 2=20-30,3=30-40,4=40-50,5=50-60,6=above 60; gender value 1=male, 2=female; ethnicity value 1=Chinese, 2=Korean Chinese, 3=Manchu, 4=Hui, 5 is others.

| **Variables** | **Description** | **Group1** | **Group 2** | **Group 3** | **Total** |
| --- | --- | --- | --- | --- | --- |
| Age | Mean value | 4.59 | 4.66 | 4.46 | 4.60 |
|  | Number of cases | 69 | 41 | 13 | 123 |
|  | Percent of total | 56.1% | 33.3% | 10.6% | 100.0% |
| Gender | Mean value | 1.66 | 1.49 | 1.50 | 1.59 |
|  | Number of cases | 70 | 41 | 12 | 123 |
|  | Percent of total | 56.9% | 33.3% | 9.8% | 100.0% |
| Ethnicity | Mean value | 1.09 | 1.07 | 1.62 | 1.14 |
|  | Number of cases | 69 | 41 | 13 | 123 |
|  | Percent of total | 56.1% | 33.3% | 10.6% | 100.0% |
| Attitudes towards tigers | Mean value | 2.66 | 4.15 | 1.08 | 2.98 |
|  | Number of cases | 70 | 41 | 13 | 124 |
|  | Percent of total | 56.5% | 33.1% | 10.5% | 100.0% |
| Attitudes towards bears | Mean value | 2.70 | 4.10 | 1.00 | 2.98 |
|  | Number of cases | 70 | 41 | 13 | 124 |
|  | Percent of total | 56.5% | 33.1% | 10.5% | 100.0% |
| Attitudes towards leopards | Mean value | 2.53 | 3.49 | 1.00 | 2.69 |
|  | Number of cases | 70 | 41 | 13 | 124 |
|  | Percent of total | 56.5% | 33.1% | 10.5% | 100.0% |
