# Supplementary material for: Community attitudes towards Amur tigers (Panthera tigris altaica) and their prey species in Yanbian, Jilin province, a region of northeast China where tigers are returning
Source: PLoS One. 2022 Oct 27;17(10):e0276554. doi: 10.1371/journal.pone.0276554 (PMC9612539; doi:10.1371/journal.pone.0276554)
Supplement: S5 Table — (DOCX) [file pone.0276554.s005.docx]

**S5 Table. Demography information in different groups of attitudes towards deer species.** In the variables, age value from 1=18-20, 2=20-30,3=30-40,4=40-50,5=50-60,6=above 60; gender value 1=male, 2=female; ethnicity value 1=Chinese, 2=Korean Chinese, 3=Manchu, 4=Hui, 5 is others.

| **Variables** | **Description** | **Group1** | **Group 2** | **Group 3** | **Total** |
| --- | --- | --- | --- | --- | --- |
| Age | Mean value | 4.71 | 4.29 | 3.91 | 4.61 |
|  | Number of cases | 104 | 7 | 11 | 122 |
|  | Percent of total | 85.2% | 5.7% | 9.0% | 100.0% |
| Gender | Mean value | 1.58 | 1.43 | 1.73 | 1.58 |
|  | Number of cases | 104 | 7 | 11 | 122 |
|  | Percent of total | 85.2% | 5.7% | 9.0% | 100.0% |
| Ethnicity | Mean value | 1.09 | 1.86 | 1.18 | 1.14 |
|  | Number of cases | 104 | 7 | 11 | 122 |
|  | Percent of total | 85.2% | 5.7% | 9.0% | 100.0% |
| Attitudes towards sika deer | Mean value | 4.00 | 1.57 | 3.64 | 3.83 |
|  | Number of cases | 105 | 7 | 11 | 123 |
|  | Percent of total | 85.4% | 5.7% | 8.9% | 100.0% |
| Attitudes towards red deer | Mean value | 3.86 | 1.57 | 3.45 | 3.69 |
|  | Number of cases | 105 | 7 | 11 | 123 |
|  | Percent of total | 85.4% | 5.7% | 8.9% | 100.0% |
| Attitudes towards roe deer | Mean value | 3.72 | 1.57 | 1.64 | 3.41 |
|  | Number of cases | 105 | 7 | 11 | 123 |
|  | Percent of total | 85.4% | 5.7% | 8.9% | 100.0% |
