# Supplementary material for: Community attitudes towards Amur tigers (Panthera tigris altaica) and their prey species in Yanbian, Jilin province, a region of northeast China where tigers are returning
Source: PLoS One. 2022 Oct 27;17(10):e0276554. doi: 10.1371/journal.pone.0276554 (PMC9612539; doi:10.1371/journal.pone.0276554)
Supplement: S6 Table — (DOCX) [file pone.0276554.s006.docx]

**S6 Table. Demography information in different groups of attitudes towards wild boars.** In the variables, age value from 1=18-20, 2=20-30,3=30-40,4=40-50,5=50-60,6=above 60; gender value 1=male, 2=female; ethnicity value 1=Chinese, 2=Korean Chinese, 3=Manchu, 4=Hui, 5 is others.

| **Variables** | **Description** | **Group1** | **Group 2** | **Group 3** | **Total** |
| --- | --- | --- | --- | --- | --- |
| Age | Mean value | 4.47 | 4.80 | 4.71 | 4.61 |
|  | Number of cases | 53 | 5 | 63 | 121 |
|  | Percent of total | 43.8% | 4.1% | 52.1% | 100.0% |
| Gender | Mean value | 1.69 | 1.80 | 1.48 | 1.59 |
|  | Number of cases | 54 | 5 | 62 | 121 |
|  | Percent of total | 44.6% | 4.1% | 51.2% | 100.0% |
| Ethnicity | Mean value | 1.15 | 1 | 1.14 | 1.14 |
|  | Number of cases | 53 | 5 | 63 | 121 |
|  | Percent of total | 43.8% | 4.1% | 52.1% | 100.0% |
| Do you often see the programs related to wildlife | Mean value | 0.5 | 0.4 | 0.51 | 0.50 |
|  | Number of cases | 54 | 5 | 63 | 122 |
|  | Percent of total | 44.3% | 4.1% | 51.6% | 100.0% |
| Attitudes towards wild boar | Mean value | 1.85 | 4 | 1.48 | 1.75 |
|  | Number of cases | 54 | 5 | 63 | 122 |
|  | Percent of total | 44.3% | 4.1% | 51.6% | 100.0% |
| The agreement to the statement’ wild boar population is too big; we should set some snares in the mountain hunt some’ | Mean value | 4.07 | 1.60 | 1.43 | 2.61 |
|  | Number of cases | 54 | 5 | 63 | 122 |
|  | Percent of total | 44.3% | 4.1% | 51.6% | 100.0% |
